# Convergent Evolution of a Promiscuous 3-Hydroxypropionyl-CoA Dehydratase/Crotonyl-CoA Hydratase in *Crenarchaeota* and *Thaumarchaeota*

**DOI:** 10.1128/mSphere.01079-20

**Published:** 2021-01-20

**Authors:** Li Liu, Philip C. Brown, Martin Könneke, Harald Huber, Simone König, Ivan A. Berg

**Affiliations:** a Institute for Molecular Microbiology and Biotechnology, University of Münster, Münster, Germany; b Department of Microbiology, Faculty of Biology, University of Freiburg, Freiburg, Germany; c Marine Archaea Group, MARUM Center for Marine Environmental Sciences, University of Bremen, Bremen, Germany; d Institute for Microbiology and Archaeal Center, Regensburg University, Regensburg, Germany; e Core Unit Proteomics, Interdisciplinary Center for Clinical Research, Medical Faculty, University of Münster, Münster, Germany; National Institute of Advanced Industrial Science and Technology

**Keywords:** 3-hydroxypropionate/4-hydroxybutyrate cycle, 3-hydroxypropionyl-CoA dehydratase, *Metallosphaera sedula*, *Nitrosopumilus maritimus*, autotrophs, crotonyl-CoA hydratase

## Abstract

The autotrophic 3-hydroxypropionate/4-hydroxybutyrate (HP/HB) cycle functions in thermoacidophilic, (micro)aerobic, hydrogen-oxidizing *Crenarchaeota* of the order *Sulfolobales* as well as in mesophilic, aerobic, ammonia-oxidizing *Thaumarchaeota.* Notably, the HP/HB cycle evolved independently in these two archaeal lineages, and crenarchaeal and thaumarchaeal versions differ regarding their enzyme properties and phylogeny. These differences result in altered energetic efficiencies between the variants. Compared to the crenarchaeal HP/HB cycle, the thaumarchaeal variant saves two ATP equivalents per turn, rendering it the most energy-efficient aerobic pathway for carbon fixation. Characteristically, the HP/HB cycle includes two enoyl coenzyme A (CoA) hydratase reactions: the 3-hydroxypropionyl-CoA dehydratase reaction and the crotonyl-CoA hydratase reaction. In this study, we show that both reactions are catalyzed in the aforementioned archaeal groups by a promiscuous 3-hydroxypropionyl-CoA dehydratase/crotonyl-CoA hydratase (Msed_2001 in crenarchaeon Metallosphaera sedula and Nmar_1308 in thaumarchaeon Nitrosopumilus maritimus). Although these two enzymes are homologous, they are closely related to bacterial enoyl-CoA hydratases and were retrieved independently from the same enzyme pool by the ancestors of *Crenarchaeota* and *Thaumarchaeota*, despite the existence of multiple alternatives. This striking similarity in the emergence of enzymes involved in inorganic carbon fixation from two independently evolved pathways highlights that convergent evolution of autotrophy could be much more widespread than anticipated.

**IMPORTANCE** Inorganic carbon fixation is the most important biosynthetic process on Earth and the oldest type of metabolism. The autotrophic HP/HB cycle functions in *Crenarchaeota* of the order *Sulfolobales* and in ammonia-oxidizing *Archaea* of the phylum *Thaumarchaeota* that are highly abundant in marine, terrestrial, and geothermal environments. Bioinformatic prediction of the autotrophic potential of microorganisms or microbial communities requires identification of enzymes involved in autotrophy. However, many microorganisms possess several isoenzymes that may potentially catalyze the reactions of the cycle. Here, we studied the enzymes catalyzing 3-hydroxypropionyl-CoA dehydration and crotonyl-CoA hydration in Nitrosopumilus maritimus (*Thaumarchaeota*) as well as in Metallosphaera sedula (*Crenarchaeota*). We showed that both reactions were catalyzed by homologous promiscuous enzymes, which evolved independently from each other from their bacterial homologs. Furthermore, the HP/HB cycle is of applied value, and knowledge of its enzymes is necessary to transfer them to a heterologous host for synthesis of various value-added products.

## Abstract (precis)

Inorganic carbon fixation is the most important biosynthetic process on Earth and the oldest type of metabolism. The autotrophic HP/HB cycle functions in *Crenarchaea* of the order *Sulfolobales* and in ammonia-oxidizing *Archaea* of the phylum *Thaumarchaeota* that are highly abundant in marine, terrestrial, and geothermal environments.

## INTRODUCTION

**FIG 1:**
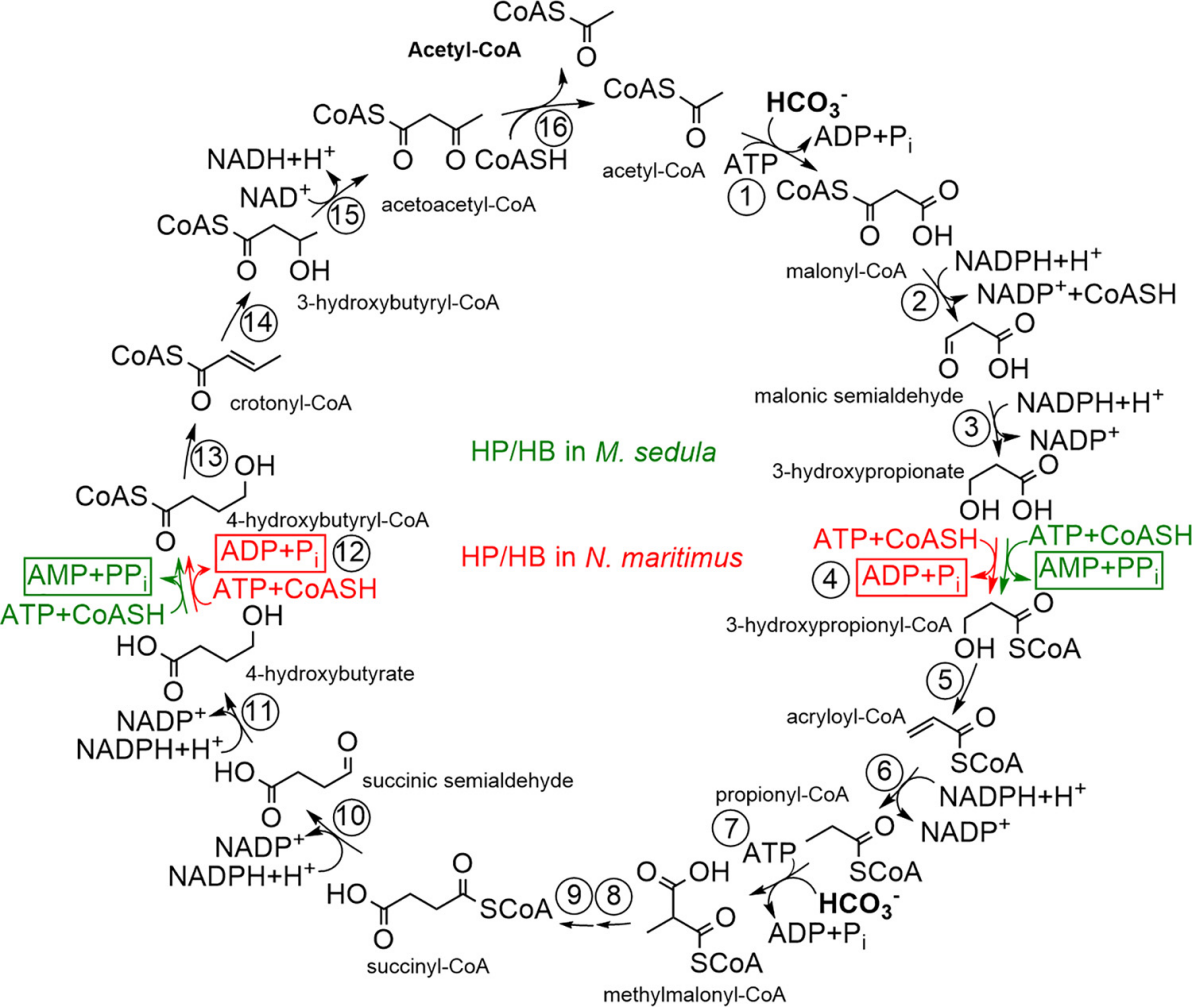
3-Hydroxypropionate/4-hydroxybutyrate cycle in *M. sedula* and *N. maritimus* (adapted from reference 13). The reactions controlling energy efficiency of the cycle in *M. sedula* are shown in green, and those for *N. maritimus* variant are shown in red. The common reactions for both are shown in black. Enzymes: 1, acetyl-CoA carboxylase; 2, malonyl-CoA reductase; 3, malonic semialdehyde reductase; 4, 3-hydroxypropionyl-CoA synthetase; 5, 3-hydroxypropionyl-CoA dehydratase; 6, acryloyl-CoA reductase; 7, propionyl-CoA carboxylase; 8, methylmalonyl-CoA epimerase; 9, methylmalonyl-CoA isomerase; 10, succinyl-CoA reductase; 11, succinic semialdehyde reductase; 12, 4-hydroxybutyryl-CoA synthetase; 13, 4-hydroxybutyryl-CoA dehydratase; 14, crotonyl-CoA hydratase; 15, (*S*)-3-hydroxybutyryl-CoA dehydrogenase; 16, acetoacetyl-CoA β-ketothiolase.

Autotrophic CO_2_ fixation is responsible for primary production, being the quantitatively most important biosynthetic process on Earth. It proceeds mainly through the Calvin-Benson cycle that evolved quite recently but became a superior autotrophic pathway due to its oxygen tolerance (1; for recent reviews, see references 2–4). However, two major anaerobic autotrophic pathways may be traced back to a hypothetical abiotic chemistry: the reductive acetyl coenzyme A (CoA) pathway and the reductive tricarboxylic acid cycle (5–7). Furthermore, the anaerobic, selenocysteine-dependent reductive glycine pathway has recently been described in Desulfovibrio desulfuricans (8). Three other known CO_2_ fixation pathways are not broadly distributed and restricted to defined phylogenetic groups: the 3-hydroxypropionate bi-cycle to *Chloroflexi*, the dicarboxylate/4-hydroxybutyrate cycle to anaerobic *Crenarchaeota*, and the 3-hydroxypropionate/4-hydroxybutyrate (HP/HB) cycle to thermoacidophilic, (micro)aerobic, hydrogen-oxidizing *Crenarchaeota* of the order *Sulfolobales* (9–12) and to oligotrophic, mesophilic, aerobic, ammonia-oxidizing *Thaumarchaeota* (13) (Fig. 1). The presence of the HP/HB cycle in two distantly related archaeal groups with different physiology was counterintuitive, as CO_2_ fixation is the central anabolic process in an autotroph and should be adapted to the corresponding ecological niche.

The biochemical and phylogenetical analysis of the enzymes of the HP/HB cycle performed in the model organisms Metallosphaera sedula (*Sulfolobales*/*Crenarchaeota*) and Nitrosopumilus maritimus (*Thaumarchaeota*) revealed that the pathway evolved independently in these two lineages and that the crenarchaeal and thaumarchaeal versions of the cycle differ in their properties (13, 14). While those enzymes that catalyze the mechanistically challenging reactions (reactions 1, 7, 9, and 13 in Fig. 1) evolved only once during evolution, and thus are phylogenetically related in these archaeal groups, other enzymes that catalyze specific reactions of the cycle (reactions 2, 3, 4, 6, 10, 11, and 12) are not homologous in *Sulfolobales* and *Thaumarchaeota*. Importantly, synthetases (reactions 4 and 12) activating 3-hydroxypropionate and 4-hydroxybutyrate produce ADP and phosphate in *Thaumarchaeota* and AMP and pyrophosphate (that is further cleaved into two phosphates) in *Sulfolobales*. The thaumarchaeal variant therefore saves two ATP equivalents in the activation of 3-hydroxypropionate and 4-hydroxybutyrate compared to the crenarchaeal variant. This is considered a crucial adaptation to the low energy yields and nutrient-poor habitats of ammonia-oxidizing archaea in *Thaumarchaeota* in contrast to those of hydrogen-oxidizing archaea in *Crenarchaeota* (13).

The pathway encompasses two enoyl-CoA hydratase reactions, i.e., dehydration of 3-hydroxypropionyl-CoA to acryloyl-CoA (3-hydroxypropionyl-CoA dehydratase) (reaction 5 in Fig. 1) and hydration of crotonyl-CoA to (*S*)-3-hydroxybutyryl-CoA (3-hydroxybutyryl-CoA dehydratase, here referred to its function as crotonyl-CoA hydratase) (reaction 14 in Fig. 1). In *M. sedula*, two dehydratases/hydratases have been characterized, (i) 3-hydroxypropionyl-CoA dehydratase (3HPD) Msed_2001 that acts equally well as crotonyl-CoA hydratase, and (ii) a bifunctional fusion protein crotonyl-CoA hydratase/(*S*)-3-hydroxybutyryl-CoA dehydrogenase Msed_0399; they are both present in, and were purified from, autotrophically grown cells (15, 16). In *M. sedula* cell extracts, correspondingly, the crotonyl-CoA hydratase activity is much higher than the 3-hydroxypropionyl-CoA hydratase activity (10, 17). However, we have recently shown that, in *M. sedula*, the (*S*)-3-hydroxybutyryl-CoA dehydrogenase reaction is primarily catalyzed by Msed_1423 (which is universally found in autotrophic *Sulfolobales*) and that the bifunctional crotonyl-CoA hydratase/(*S*)-3-hydroxybutyryl-CoA dehydrogenase Msed_0399 is apparently not the main crotonyl-CoA hydratase in this archaeon, although this protein is present in autotrophically grown cells (17). Indeed, the genome contains four more dehydratase candidates that could catalyze crotonyl-CoA hydratase reaction. In contrast, *N. maritimus* contained only one dehydratase candidate, a promiscuous 3-hydroxypropionyl-CoA dehydratase/crotonyl-CoA hydratase, which was shown to operate with both substrates (13).

Therefore, we reevaluated the identity of crotonyl-CoA hydratase and compared the corresponding enzymes in *N. maritimus* and *M. sedula*. Surprisingly, the enzyme characterization and quantification of various crotonyl-CoA hydratases in *M. sedula* performed here revealed that the previously characterized 3-hydroxypropionyl-CoA dehydratase Msed_2001 is mainly responsible for the crotonyl-CoA hydratase reaction *in vivo*, rendering it a promiscuous enzyme, as is the case for *N. maritimus*. Furthermore, our phylogenetic analysis showed that, although both *M. sedula* and *N. maritimus* enzymes evolved independently from different bacterial enoyl-CoA hydratases, they are highly homologous, which reveals striking similarities in the course of convergent evolution of autotrophic CO_2_ fixation.

## RESULTS

### Crotonyl-CoA hydratase and 3-hydroxypropionyl-CoA dehydratase reactions in *M. sedula* cell extracts.

**FIG 2:**
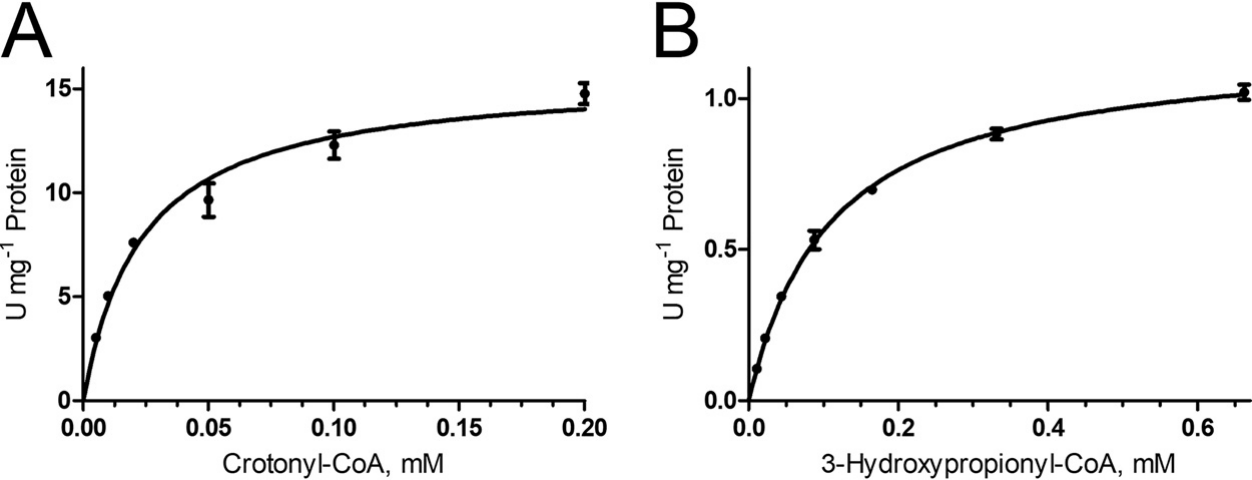
Kinetics of crotonyl-CoA hydratase (A) and 3-hydroxypropionyl-CoA dehydratase (B) reactions in extracts of autotrophically grown *M. sedula*. The determined *V*_max_ and *K_m_* values were: (A) 15.7 ± 0.6 U mg^−1^ protein and 23 ± 3 μM for crotonyl-CoA and (B) 1.18 ± 0.02 U mg^−1^ protein and 109 ± 5 μM for 3-hydroxypropionyl-CoA.

The HP/HB cycle contains two enoyl-CoA hydratase reactions, the 3-hydroxypropionyl-CoA dehydratase and the crotonyl-CoA hydratase reaction. The study of their kinetics in cell extracts of autotrophically grown *M. sedula* showed that they followed Michaelis-Menten kinetics with *V*_max_ of 15.7 ± 0.6 U mg^−1^ protein and *K_m_* of 23 ± 3 μM for crotonyl-CoA hydration (65°C) and 1.18 ± 0.02 U mg^−1^ protein and 109 ± 5 μM for 3-hydroxypropionyl-CoA dehydration (42°C) (Fig. 2). Usually, reactions catalyzed by several enzymes do not follow Michaelis-Menten kinetics, which suggests that each of the corresponding reactions is catalyzed by a single enzyme and not by several isoenzymes. An array of proteins could be responsible for these activities: *M. sedula* genome harbors six crotonyl-CoA hydratase homologs (Table 1), and four of them were previously characterized. One of these homologs was identified as 3-hydroxypropionyl-CoA dehydratase (Msed_2001) (15, 18). This protein is universally conserved among autotrophic *Sulfolobales* (Table 1). Uncharacterized crotonyl-CoA hydratase homolog Msed_0566 was also present in all autotrophic *Crenarchaeota*, while four other homologs were present only in some strains. Based on this fact, it was proposed that Msed_0566 may be a designated crotonyl-CoA hydratase in the crenarchaeal HP/HB cycle (17).

**TABLE 1:** Properties of the crotonyl-CoA hydratase homologs in *M. sedula*, based on the published data^*e*^

Substrate, parameter	Msed_2001^*a*^^,^^*b*^	Msed_0399^*c*^^,^^*d*^	Msed_0336^*c*^	Msed_0384^*c*^	Msed_0385^*c*^	Msed_0566^*c*^
3-Hydroxypropionyl-CoA
*V*_max_, U mg^−1^ protein	372^*a*^/544^*b*^	4.8	4 (Sp. act.)	4 (Sp. act.)	ND	ND
*K_m_*, mM	0.06^*a*^/0.025^*b*^	0.06	ND	ND		
*k*_cat_/*K_m_*, s^−1 ^mM^−1^	3,190^*a*^/10,260^*b*^	94	NA	NA		
Crotonyl-CoA
*V*_max_, U mg^−1^ protein	ND	526	52	454	ND	ND
*K_m_*, mM		0.97	0.08	0.22		
*k*_cat_/*K_m_*, s^−1 ^mM^−1^		640	317	1,043		
(*S*)-3-Hydroxybutyryl-CoA
*V*_max_, U mg^−1^ protein	385^*a*^	246	119	242	ND	ND
*K_m_*, mM	0.075^*a*^	0.86	0.07	0.05		
*k*_cat_/*K_m_*, s^−1 ^mM^−1^	2,640^*a*^	338	830	2,445		
Presence in all autotrophic *Sulfolobales*^*c*^	Yes	No	No	No	No	Yes

a The protein was purified from *M. sedula* cell extracts and characterized as 3-hydroxypropionyl-CoA hydratase; data from reference 15.

b Data from reference 18

c Data from reference 17.

d Bifunctional crotonyl-CoA hydratase/3-hydroxybutyryl-CoA dehydrogenase Msed_0399 is a fusion protein consisting of an enoyl-CoA hydratase domain and a dehydrogenase domain.

e The *V*_max_ values were extrapolated to 75°C based on the assumption that a 10°C rise in temperature doubles the reaction rate. ND, not determined; NA, not applicable.

### Characterization of an enoyl-CoA hydratase Msed_0566.

The gene *msed_0566* was cloned and heterologously expressed in Escherichia coli, and the corresponding protein was studied biochemically. Although it was active with the tested enoyl-CoAs, the activity was low (Table 2). Previous attempts to characterize the heterologously produced Msed_0566 were unsuccessful (17), probably due to the low expression level of the protein and its poor activity with crotonyl-CoA. Bad catalytic efficiency (*k*_cat_/*K_m_*) with the tested substrates suggests that the protein is specific to unknown substrate(s) and is not involved in the HP/HB cycle. The question remains as to which of the enoyl-CoA hydratase homologs is predominantly responsible for crotonyl-CoA conversion into (*S*)-3-hydroxybutyryl-CoA in *M. sedula*. Considering that no genetic system is available for *M. sedula*, we decided to study the abundance of the corresponding enoyl-CoA hydratase homologs in extracts of autotrophically grown *M. sedula* cells using a proteomic approach in order to evaluate their importance in autotrophic metabolism.

**TABLE 2:** Catalytic properties of putative crotonyl-CoA hydratases Msed_0566^*d*^

Substrate	*V*_max_ (U mg^−1^ protein)		*K_m_* (mM)	*k*_cat_/*K_m_* (s^−1^ mM^−1^)^*a*^
	Measured (65°C)	Extrapolated to 75°C		
**Crotonyl-CoA**	1.37 ± 0.15	2.74 ± 0.30	0.08 ± 0.03	19
**(*S*)-3-Hydroxybutyryl-CoA**	0.07^*b*^*^,^*^*c*^	0.7	NA	NA
**3-Hydroxypropionyl-CoA**	0.04^*b*^*^,^*^*c*^	0.4	NA	NA
**Acryloyl-CoA**	1.34 ± 0.04	2.68 ± 0.08	0.037 ± 0.002	39
**Methacrylyl-CoA**	0.26 ± 0.02	0.52 ± 0.04	0.14 ± 0.03	2
**(*E*)-2-Octenoyl-CoA**	2.61 ± 0.34	5.22 ± 0.68	0.18 ± 0.06	16

a *k*_cat_ was calculated for the activities at 75°C.

b Activity was measured at 42°C.

c Specific activity.

d The *V*_max_ values are normalized to 75°C based on the assumption that a 10°C rise in temperature doubles the reaction rate. NA, not applicable.

### Quantification of enoyl-CoA hydratase homologs in autotrophically grown *M. sedula*.

The concentrations of the six enoyl-CoA hydratase homologs were assessed using data-independent mass spectrometric and ion mobility analysis of the total proteome tryptic digest. We found that all six proteins were present in autotrophically grown *M. sedula* and that their abundance can be ranked in the order Msed_2001 (set to 100%) > Msed_0399 (89%) > Msed_0384 (68%) > Msed_0385 (47%) > Msed_0566 (42%) > Msed_0336 (16%) (Table 3). The characterized crotonyl-CoA hydratase homologs (Msed_0399, Msed_0384, Msed_0336, and Msed_0566) display a *V*_max_ too low to be responsible for the reaction *in vivo*. Indeed, the comparison of *V*_max_ values measured for each of the characterized enzymes and for *M. sedula* cell extracts resulted in much higher estimated abundances (6.0, 6.9, 60.4, and >100%, respectively; compare Tables 1 and 2 and Fig. 2). Despite all our attempts, we could not produce Msed_0385 heterologously in a soluble and active form. Nevertheless, this protein is not universally conserved in autotrophic *Sulfolobales* and thus does not appear to be the sought-after crotonyl-CoA hydratase. Therefore, we decided to reevaluate the function of the 3-hydroxypropionyl-CoA dehydratase Msed_2001 (which is present in all autotrophic *Sulfolobales*; reference 17) and to characterize this protein with different substrates.

**TABLE 3:** Quantification of enoyl-CoA hydratase homologs in cell extracts of autotrophically grown *M. sedula* using high-definition mass spectrometry^*a*^

Protein	Relative protein concentration, %	Comparison to Msed_2001, %
Msed_2001	0.19	100
Msed_0399	0.17	89
Msed_0336	0.03	16
Msed_0384	0.13	68
Msed_0385	0.09	47
Msed_0566	0.08	42

a Samples were spiked with known amounts of yeast alcohol dehydrogenase 1 and enolase 1, and proteins were quantified in reference to the total protein amount (ng); the average is given.

### Msed_2001 is a promiscuous 3-hydroxypropionyl-CoA dehydratase/crotonyl-CoA hydratase.

The gene *msed_2001* was cloned in vector pET16b and heterologously expressed, and the corresponding protein Msed_2001 was purified and characterized. As expected, it was highly active with 3-hydroxypropionyl-CoA (Table 4), which was already shown to be its natural substrate (15, 18). The measured *V*_max_ and *K_m_* values of Msed_2001 to 3-hydroxypropionyl-CoA were close to those determined previously (see Table 1). Surprisingly, the protein was also highly active with all other tested substrates (Table 4). In particular, it had a very high *V*_max_ value to crotonyl-CoA. Although the same reaction was detected with a lower *K_m_* value in cell extracts, the difference was in the range of deviation, which can be expected of heterologously produced enzymes. Importantly, the ratio of crotonyl-CoA hydratase and 3-hydroxypropionyl-CoA dehydratase activities in cell extracts (3:1) was close to that of Msed_2001 (5:1). Considering a universal distribution of this protein in autotrophic *Sulfolobales*, we conclude that Msed_2001 is a promiscuous 3-hydroxypropionyl-CoA dehydratase/crotonyl-CoA hydratase that is responsible for both conversions *in vivo*. Msed_0399, which was previously thought to be responsible for crotonyl-CoA conversion into acetoacetyl-CoA, is responsible for a small flux in autotrophically grown *M. sedula*, at best.

**TABLE 4:** Catalytic properties of 3-hydroxypropionyl-CoA dehydrases/crotonyl-CoA hydratases Msed_2001 and Nmar_1308 as well as homologous enoyl-CoA hydratase Slip_2089 from *S. lipocalidus*^*d*^

Substrate	Msed_2001				Nmar_1308			Slip_2089			
	*V*_max_ (U·mg^−1^ protein)		*K_m_* (mM)	*k*_cat_/*K_m_* (s^−1^ mM^−1^)^*a*^	*V*_max_ (U·mg^−1^ protein)	*K_m_* (mM)	*k*_cat_/*K_m_* (s^−1^ mM^−1^)^*a*^	*V_max_* (U·mg^−1^ protein)		*K_m_* (mM)	*k*_cat_/*K_m_* (s^−1^ mM^−1^)^*c*^
	Measured (65°C)	Extrapolated to 75°C						Measured (42°C)	Extrapolated to 55°C		
**Crotonyl-CoA**	2,461 ± 135	4,922 ± 270	0.08 ± 0.01	31,621	3,793 ± 111	0.45 ± 0.03	4,251	18 ± 1	44 ± 2	0.16 ± 0.02	143
**(*S*)-3-Hydroxybutyryl-CoA**	294 ± 11^*b*^	2,896 ± 108	0.034 ± 0.004	43,776	1,515 ± 92	0.36 ± 0.06	2,122	42 ± 5	103 ± 12	1.31 ± 0.31	41
**3-Hydroxypropionyl-CoA**	103 ± 5^*b*^	1,014 ± 49	0.16 ± 0.02	3,257	38 ± 2	0.17 ± 0.03	113	0.45 ± 0.03	1.11 ± 0.07	0.35 ± 0.06	2
**Acryloyl-CoA**	13,532 ± 572	27,064 ± 1,144	0.14 ± 0.01	99,354	11,391 ± 392	0.71 ± 0.07	8,091	43 ± 2	106 ± 5	0.40 ± 0.05	138
**Methacrylyl-CoA**	804 ± 43	1,608 ± 86	2.19 ± 0.23	377	116 ± 13	0.83 ± 0.24	70	0.38 ± 0.03	0.94 ± 0.07	1.01 ± 0.22	0.5
**(*E*)-2-Octenoyl-CoA**	1,842 ± 70	3,684 ± 140	0.048 ± 0.005	39,446	47 ± 2	0.12 ± 0.02	198	92 ± 5	227 ± 12	0.21 ± 0.04	562

a *k*_cat_ was calculated for the activities at 75°C.

b Activity was measured at 42°C.

c *k*_cat_ was calculated for the activities at 55°C.

d The *V*_max_ values are normalized to 75°C based on the assumption that a 10°C rise in temperature doubles the reaction rate.

Interestingly, the functioning of a promiscuous 3-hydroxypropionyl-CoA dehydratase/crotonyl-CoA hydratase (Nmar_1308) was also proposed for the HP/HB cycle in *N. maritimus*, which was thought to be the result of convergent evolution with no relation to the *M. sedula* cycle (13). As Nmar_1308 is the only enoyl-CoA hydratase homolog in *N. maritimus*, it was obvious from the beginning that a promiscuous enzyme catalyzes both reactions, being in sharp contrast to what was known for *M. sedula* at that time. To compare the corresponding enzymes in *Crenarchaeota* and *Thaumarchaeota*, we thoroughly characterized Nmar_1308.

### Characterization of 3-hydroxypropionyl-CoA dehydratase/crotonyl-CoA hydratase Nmar_1308 from *N. maritimus* and its phylogenetic analysis.

*N. maritimus nmar_1308* was cloned in the vector pET16b and heterologously produced in E. coli. The purified protein was highly active in both 3-hydroxypropionyl-CoA dehydratase and crotonyl-CoA hydratase reactions and had catalytic properties very similar to those of *M. sedula* protein (Table 4). Both proteins indeed share notable sequence similarity (42/69% of sequence identity/similarity); hence, phylogenetic analysis was performed.

**FIG 3:**
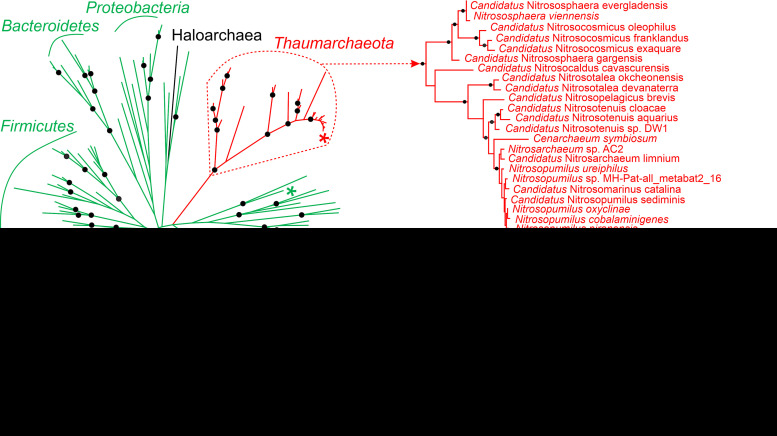
Maximum likelihood phylogenetic tree of 3-hydroxypropionyl-CoA dehydratases/crotonyl-CoA hydratases. Msed_2001, Nmar_1308, and Slip_2089 are marked with *. Bacterial sequences are shown in green, euryarchaeal in black, the sequences of ammonia-oxidizing *Thaumarchaeota* in red, and sequences of non-ammonia-oxidizing (non-AOA) *Thaumarchaeota* in blue. The scale bar represents a difference of 0.2 substitutions per site. The percentage bootstrap values for the clade of this group were calculated in 1,000 replications. Only values above 70% are shown (shown as ●). The accession numbers are listed in Table S3. The tree constructed by maximum-parsimony algorithm (data not shown) was similar with minor exceptions.

The homologs of *N. maritimus* protein were found in all ammonia-oxidizing *Thaumarchaeota* sequenced so far (Table S1). Interestingly, phylogeny of 3-hydroxypropionyl-CoA dehydratase/crotonyl-CoA hydratase fits well to the current taxonomy of this group (Fig. 3), suggesting that the gene was transferred vertically during its evolution and was most probably present in the ancestor of ammonia-oxidizing *Thaumarchaeota*. Thaumarchaeal 3-hydroxypropionyl-CoA dehydratase/crotonyl-CoA hydratases are closely related to a number of bacterial proteins and build a separate phylogenetic branch on the tree. The genomes of *Thaumarchaeota* that are not able to oxidize ammonia (and apparently do not grow autotrophically) possess Nmar_1308 homologs as well. However, they do not cluster with Nmar_1308 in the tree and probably evolved independently from them (Fig. 3). Furthermore, thaumarchaeal 3-hydroxypropionyl-CoA dehydratase/crotonyl-CoA hydratases are only distantly related to Msed_2001 homologs present in *Sulfolobales* (Fig. 3), revealing that both proteins evolved independently from phylogenetically related bacterial homologs. Hence, catalytic properties of one of those homologs, i.e., the enoyl-CoA hydratase homolog from Syntrophothermus lipocalidus Slip_2089, were studied.

10.1128/mSphere.01079-20.1 TABLE S1 Distribution of the known *N. maritimus* genes encoding enzymes of the HP/HB cycle in fully sequenced *Thaumarchaeota* as well as in several non-ammonia-oxidizing (non-AOA) *Thaumarchaeota* (according to Fig. 1 of reference 1). The accession numbers or the sequences of the *N. maritimus* proteins used as BLASTP query sequences are shown (Nmar_####). Download Table S1, XLSX file, 0.02 MB. Copyright © 2021 Liu et al. 2021 Liu et al. This content is distributed under the terms of the Creative Commons Attribution 4.0 International license.

### Characterization of a putative crotonyl-CoA hydratase Slip_2089 from *S. lipocalidus*.

Although the heterologously produced Slip_2089 was active at best with (*E*)-2-octenoyl-CoA, it also possessed noticeable activity with both crotonyl-CoA and 3-hydroxypropionyl-CoA (Table 4). These activities are relatively low and probably not physiologically relevant for *S. lipocalidus*. Nevertheless, their presence appears to be a common property of enoyl-CoA hydratases of this family, suggesting that the ancestral proteins for both groups possessed the two required activities and were well suited to ever function in the HP/HB cycle and to adapt to the performed functions.

## DISCUSSION

Although both *Crenarchaeota* and *Thaumarchaeota* belong to the TACK superphylum (named after *Thaum-*, *Aig-*, *Cren-*, and *Korarchaeota*), they are only distant relatives on the phylogenetic tree. Furthermore, *M. sedula* (representative of *Sulfolobales*) and *N. maritimus* (representative of ammonia-oxidizing *Thaumarchaeota*) are adapted to very different ecological niches. *Sulfolobales* are (hyper)thermophilic and acidophilic archaea that prefer (micro)aerobic conditions. Many strains are able to grow autotrophically, using elemental sulfur, thiosulfate, sulfidic ores, or molecular hydrogen for energy generation (19). In contrast, ammonia-oxidizing archaea of the phylum *Thaumarchaeota* are mesophilic to thermophilic, mainly autotrophic archaea known for their unusually high affinity to ammonia (in the low nanomolar range, as studied in *N. maritimus*) (20), being thus adapted to lower energy supply than bacterial ammonia oxidizers. Our analysis of the distribution of genes encoding enzymes of the HP/HB cycle revealed their occurrence in all genomes of ammonia-oxidizing *Thaumarchaeota* sequenced so far but their absence in nonautotrophic, non-ammonia-oxidizing members of the phylum (Table S1). This finding corroborates the results of recent ancestral genomic reconstructions dating the emergence of the thaumarchaeal HP/HB cycle back to the last common ancestor of ammonia-oxidizing *Thaumarchaeota* (21). Interestingly, some non-ammonia-oxidizing *Thaumarchaeota* possess an enoyl-CoA hydratase homologous to Msed_2001 (Fig. 3). Its function in these heterotrophic archaea is unknown.

Corresponding to the difference in the physiology, *Sulfolobales* and *Thaumarchaeota* use different variants of the HP/HB cycle. In these two variants, the enzymes catalyzing specific conversions of the cycle, i.e., reductions of malonyl-CoA to propionyl-CoA (reactions 2 to 4 and 6 in Fig. 1) and of succinyl-CoA to 4-hydroxybutyryl-CoA (reactions 10 to 12), are unique and not homologous to each other (13, 14). Acetyl-CoA/propionyl-CoA carboxylase, methylmalonyl-CoA mutase, and 4-hydroxybutyryl-CoA dehydratase catalyze mechanistically challenging reactions and are broadly distributed in the microbial world. They appear to have been “invented” only once in the evolution and are understandably homologous in *Sulfolobales* and *Thaumarchaeota*. The same seems to be the case for methylmalonyl-CoA epimerase catalyzing a relatively simple deprotonation/protonation reaction (22). This enzyme emerged just once in the evolution and is homologous in *Crenarchaeota* and *Thaumarchaeota.* Nevertheless, thaumarchaeal and crenarchaeal enzymes do not build sister groups in the phylogenetic trees of the corresponding proteins and are only distantly related to each other, confirming that the HP/HB cycle was not inherited from the common ancestor (13).

The conversion of crotonyl-CoA to acetoacetyl-CoA was originally attributed to very different enzymes in *N. maritimus* and *M. sedula*. The annotation of *N. maritimus* genes was straightforward, as this organism has a relatively small, streamlined genome (1.65 Mbp [23]) that harbors only one homolog of an enoyl-CoA hydratase possessing both crotonyl-CoA hydratase and 3-hydroxypropionyl-CoA dehydratase activities and one distinct homolog of (*S*)-3-hydroxybutyryl-CoA dehydrogenase (13) (Table 4). In contrast, *M. sedula* genome encodes multiple enoyl-CoA hydratase and hydroxyacyl-CoA dehydrogenase homologs. Nevertheless, it was first assumed that the corresponding reactions are catalyzed in *M. sedula* by a bifunctional crotonyl-CoA hydratase/(*S*)-3-hydroxybutyryl-CoA dehydrogenase Msed_0399 (16, 18, 24). Indeed, the homologous fusion protein consisting of an enoyl-CoA hydratase and a dehydrogenase domain is responsible for the corresponding conversion in the dicarboxylate/4-hydroxybutyrate cycle in anaerobic *Crenarchaeota* like Ignicoccus hospitalis (*Desulfurococcales*) or Pyrobaculum neutrophilum (*Thermoproteales*) (25, 26), and it was logical to propose the same for *M. sedula*. However, we have recently shown that the (*S*)-3-hydroxybutyryl-CoA dehydrogenase reaction is catalyzed in *M. sedula* by a distinct enzyme (17). Here we confirm that Msed_0399 is not the main protein responsible for the crotonyl-CoA hydratase reaction. Moreover, we show that *M. sedula* uses a promiscuous 3-hydroxypropionyl-CoA dehydratase/crotonyl-CoA hydratase, exactly as in *N. maritimus*. Although it was known before that this protein possesses (*S*)-3-hydroxybutyryl-CoA dehydratase activity (15, 16, 18), it was not identified as the main protein responsible for the aforementioned conversion in *M. sedula in vivo* until now. In fact, that activity was attributed to another protein.

Heterologous expression often results in production of proteins with properties not identical to those of the native enzymes. Indeed, based on the *V*_max_ of crotonyl-CoA hydratase in cell extract (15.7 U mg^−1^ at 65°C) and the Msed_2001 abundance (0.19%), the expected *V*_max_ of Msed_2001 should be ∼8,300 U mg^−1^ protein (at 65°C), i.e., 3.4-fold higher than the value measured with heterologously produced Msed_2001. The *K_m_* value of the protein produced in *M. sedula* was also higher. Additional enoyl-CoA hydratases were present in *M. sedula* cell extracts (Table 3), which, too, may contribute to the crotonyl-CoA hydratase reaction in *M. sedula.* Nevertheless, their contribution is not high, as most of them have low activity. Furthermore, they are not universally conserved among autotrophic members of the *Sulfolobales*. Based on the comparison of *V*_max_ of the heterologously produced proteins (Tables 1, 2, and 4) and their abundances (Table 3), Msed_2001 is responsible for 99% of 3-hydroxypropionyl-CoA dehydratase activity and for 86% of crotonyl-CoA hydratase activity in *M. sedula*, the latter probably with some contribution of Msed_0399 (8.2%) and Msed_0384 (5.5%) enoyl-CoA hydratases (Tables 1 and 3).

Our work showed that enzymes catalyzing enoyl-CoA hydratase reactions in *Crenarchaeota* and *Thaumarchaeota* were retrieved from the same enzyme pool, despite the existence of multiple alternatives. Both enzymes are closely related to bacterial enoyl-CoA hydratases, yet cluster with different bacterial proteins and do not show a direct relationship to each other (Fig. 3). Although the physiological function of the corresponding bacterial homologs is not known, it appears that these proteins possess both 3-hydroxypropionyl-CoA dehydratase and crotonyl-CoA hydratase activities, as was shown for *S. lipocalidus* enoyl-CoA hydratase (Table 4). Their adaptation to a new function in *Thaumarchaeota*/*Sulfolobales* did not require many amino acid substitutions. Indeed, Nmar_1308 and the closest bacterial homolog (according to the BLASTP search) share 85/92% of sequence identity/similarity. A previous structural and phylogenetic analysis showed that crenarchaeal 3-hydroxypropionyl-CoA dehydratase/crotonyl-CoA hydratase homologs have an enoyl-group binding pocket similar to that of bacterial short-chain enoyl-CoA hydratases (27). This striking similarity in the evolutionary routes, leading to the emergence of similar (and homologous) enzymes that participate in two independently evolved pathways, highlights that convergent evolution of autotrophy could be much more widespread than anticipated.

## MATERIALS AND METHODS

### Materials.

Chemicals and biochemicals were obtained from Sigma-Aldrich (Deisenhofen, Germany), Fluka (Neu-Ulm, Germany), Applichem (Darmstadt, Germany), Merck (Darmstadt, Germany), VWR (Darmstadt, Germany), Roth (Karlsruhe, Germany), IBA (Göttingen, Germany), or Cell Signaling Technology (Frankfurt, Germany). Materials for cloning and expression were purchased from New England Biolabs (Frankfurt, Germany), Bioline (London, UK), MBI Fermentas (St. Leon-Rot, Germany), or Novagen (Schwalbach, Germany). Materials and equipment for protein purification were obtained from Macherey-Nagel (Düren, Germany), Thermo Scientific (Rockford, Illinois, USA), Pall Corporation (Dreieich, Germany), GE Healthcare (Freiburg, Germany), or Millipore (Eschborn, Germany). Primers were synthesized by Sigma-Aldrich (Steinheim, Germany).

### Microbial strains and growth conditions.

Cells of *M. sedula* TH2^T^ (DSM 5348) were cultivated autotrophically at 75°C in a 100-liter fermenter using modified ALLEN medium, pH 2.0 (19), under gassing with a mixture of 19% CO_2_, 3% O_2_, and 78% H_2_ (0.5 liters/min) (generation time, 8 h) (28, 29). Cell pellets were stored at −80°C until use. *N. maritimus* strain SCM1 was isolated by Martin Könneke from a fish tank of the Seattle Aquarium (30) and has since been maintained as reference strain in his laboratory using culture conditions described in reference 31. To obtain the biomass for enzymatic assays, *N. maritimus* was cultivated at 28°C in 15-liter HEPES-buffered medium (pH 7.5) as described previously (32). Cells were stored at −20°C. E. coli strain TOP10, E. coli NEB DH5α, and E. coli strain Rosetta 2 (DE3) (Merck, Darmstadt, Germany) were cultivated at 37°C in lysogeny broth (LB) medium. *S. lipocalidus* TGB-C1 (DSM 12680) DNA was obtained from Deutsche Sammlung von Mikroorganismen und Zellkulturen (DSMZ).

### CoA-esters synthesis.

Crotonyl-CoA and methacrylyl-CoA were chemically synthesized from the corresponding anhydrides and CoA (33). 3-Hydroxypropionyl-CoA was enzymatically synthesized with recombinant propionate CoA-transferase from Clostridium propionicum (34). Acryloyl-CoA was enzymatically synthesized with recombinant acyl-CoA oxidase 4 from Arabidopsis thaliana as described in Schwander et al. (35). (*S*)-3-Hydroxybutyryl-CoA and (*E*)-2-octenoyl-CoA were synthesized from the corresponding free acids by the mixed anhydride method (36) and then were purified using high-performance liquid chromatography (37).

### Preparation of *M. sedula* cell extracts.

Frozen cells (100 to 150 mg) were suspended in 0.6 ml of 20 mM Tris-HCl (pH 7.8), 5 mM dithioerythritol (DTE) containing 0.1 mg ml^−1^ DNase I in 1.5-ml Eppendorf vials. After addition of 1 g of glass beads (diameter 0.1 to 0.25 mm), the cell suspensions were treated in the mixer-mill (type Mikro-Dismembrator S, Sartorius, Göttingen, Germany) for 10 min at 1,800 rpm and the cell lysates were centrifuged for 20 min (14,000 rpm, 4°C). The supernatant (cell extract) was used for enzyme assays immediately or stored at −80°C until use.

### Gene cloning.

Primers and restriction enzymes used for the cloning of genes are listed in Table S2. The *M. sedula* gene encoding 3-hydroxypropionyl-CoA dehydratase (Msed_2001) was inserted into the expression vector pET16b with an N-terminal His_10_-tag by restriction-free cloning method (38). The *M. sedula* genes encoding enoyl-CoA hydratase (Msed_0566 and Msed_0385) and the *S. lipocalidus* gene encoding a putative crotonyl-CoA hydratase (Slip_2089) were amplified using Q5 polymerase (NEB, Frankfurt, Germany). The gene encoding 3-hydroxypropionyl-CoA dehydratase/crotonyl-CoA hydratase (Nmar_1308) was amplified from *N. maritimus* genomic DNA using MangoMix polymerase (Bioline, London, UK). The PCR products were digested with the corresponding restrictases (Table S2). The genes *msed_0566*, *msed_0385*, and *nmar_1308* were ligated into pET16b using T4 DNA ligase (NEB). The gene *slip_2089* was inserted into pET16b by means of Gibson cloning (39). The Gibson Assembly Cloning kit (NEB) was used according to the manufacturer’s instruction. Amplification of the plasmids was performed in E. coli TOP10.

10.1128/mSphere.01079-20.2 TABLE S2 Primers used in this study. The restriction enzymes used for the cloning are shown in parentheses and the corresponding restriction sites are underlined. Download Table S2, DOCX file, 0.02 MB. Copyright © 2021 Liu et al. 2021 Liu et al. This content is distributed under the terms of the Creative Commons Attribution 4.0 International license.

10.1128/mSphere.01079-20.3 TABLE S3 The GenBank accession numbers or protein sequences used for the construction of the phylogenetic tree shown in Fig. 3. Download Table S3, XLSX file, 0.02 MB. Copyright © 2021 Liu et al. 2021 Liu et al. This content is distributed under the terms of the Creative Commons Attribution 4.0 International license.

### Heterologous expression in E. coli.

E. coli Rosetta 2 (DE3) was transformed with the recombinant vectors. The cells were grown at 37°C in LB medium with 100 μg ampicillin ml^−1^ and 34 μg chloramphenicol ml^−1^. Expression of Msed_2001 was induced at OD_578_ of 0.7 with 0.5 mM isopropyl-β-d-thiogalactopyranoside (IPTG), and the temperature was lowered to 30°C. The cells were harvested after additional growth for 6 h. Expression of Msed_0566 and Msed_0385 were induced at OD_578_ of 0.6 to 0.8 with 0.2 mM IPTG, and the temperature was lowered to 16°C. The cells were harvested after additional growth for 15 h. Expression of Nmar_1308 and Slip_2089 were induced at OD_578_ of 0.6 to 0.7 with 1 mM IPTG, and the temperature was lowered to 20°C. The cells were harvested after additional growth for 3 h.

### Purification of recombinant proteins.

The heterologously produced His_10_-tagged Msed_2001, Msed_0566, Msed_0385, Nmar_1308, and Slip_2089 were purified using affinity chromatography. (i) Preparation of cell extract. Frozen E. coli cells were resuspended 1:3 (wet weight/volume) in 20 mM Tris-HCl (pH 7.8), 5 mM DTE containing 0.1 mg ml^−1^ DNase I. The suspensions were passed through a chilled French pressure cell twice at 137 MPa, and the cell lysates were centrifuged for 1 h (100,000 × *g*, 4°C). (ii) Heat precipitation. The supernatant of Msed_2001 cell lysate was incubated for 15 min at 85°C and then cooled on ice for 15 min, followed by centrifugation (14,000 rpm) at 4°C for 15 min. (iii) Affinity chromatography. The supernatants of Msed_2001, Nmar_1308, and Slip_2089 were applied at a flow rate of 0.5 ml min^−1^ to 1-ml Protino Ni-NTA columns (Macherey-Nagel), which had been equilibrated with 20 mM Tris-HCl containing 100 mM KCl (pH 7.8). The columns were washed with the same buffer containing 100 mM imidazole at a flow rate of 0.5 ml min^−1^ to elute unwanted protein. The enzymes were eluted with the same buffer containing 500 mM imidazole. The recombinant Msed_0566 and Msed_0385 were applied to 0.2-ml HisPur Ni-NTA Spin Columns (Thermo Scientific, Rockford, Illinois, USA) that had been equilibrated with 50 mM Tris-HCl containing 500 mM KCl (pH 8.0). The unwanted proteins were washed out with the same buffer containing 100 mM imidazole, and the enzymes were eluted with the same buffer containing 500 mM imidazole. The recombinant proteins were stored in 50% glycerol at −20°C after concentration using 10K Vivaspin Turbo 4 (Sartorius, Göttingen, Germany).

### Enzyme assays.

The 3-hydroxypropionyl-CoA dehydratase and 3-hydroxybutyryl-CoA dehydratase activities of Msed_2001, Msed_0566, and Msed_0385, as well as those in cell extracts of *M. sedula*, were measured at 42°C. Their other enzyme activities were measured at 65°C. All enzyme activities of Nmar_1308 and Slip_2089 were measured at 30°C and 42°C, respectively. The reactions were started by the addition of pure enzyme and were stopped after 1 min by the addition of 1 M HCl-10% acetonitrile (20 μl). Protein was removed by centrifugation (14,000 rpm) at 4°C for 20 min, and the products were analyzed by ultra-high-performance liquid chromatography (UHPLC) using a reverse-phase (RP) C_18_ column as described previously (17).

Crotonyl-CoA hydratase activity was detected by the formation of 3-hydroxybutyryl-CoA from crotonyl-CoA. The 20-μl reaction mixture contained 100 mM Tris-HCl (pH 7.8), 0.5 mM crotonyl-CoA, and purified enzyme. The concentration of crotonyl-CoA was varied (0.005 to 2 mM) for *K_m_* determination.

3-Hydroxypropionyl-CoA dehydratase was detected as 3-hydroxypropionyl-CoA-dependent acryloyl-CoA formation by coupling the reaction to crotonyl-CoA carboxylase/reductase (Ccr) from Rhodobacter sphaeroides which reductively carboxylates acryloyl-CoA and crotonyl-CoA to methylmalonyl-CoA and ethylmalonyl-CoA (40). The 20-μl reaction mixture contained 100 mM Tris-HCl (pH 7.8), 1 mM NADPH, 50 mM NaHCO_3_, 0.1 mg ml^−1^ Ccr, 0.5 mM 3-hydroxypropionyl-CoA, and purified enzyme. The concentration of 3-hydroxypropionyl-CoA was varied (0.01 to 1.5 mM) for *K_m_* determination.

3-Hydroxybutyryl-CoA dehydratase activity was detected by coupling the reaction to Ccr. The 20-μl reaction mixture contained 100 mM Tris-HCl (pH 7.8), 1 mM NADPH, 50 mM NaHCO_3_, 0.1 mg ml^−1^ Ccr, 0.2 mM 3-hydroxybutyryl-CoA, and purified enzyme. The concentration of 3-hydroxybutyryl-CoA was varied (0.005 to 2 mM) for *K_m_* determination.

Acryloyl-CoA hydratase activity was detected by the formation of 3-hydroxypropionyl-CoA from acryloyl-CoA. The 20-μl reaction mixture contained 100 mM Tris-HCl (pH 7.8), 0.1 mM acryloyl-CoA, and purified enzyme. The concentration of acryloyl-CoA was varied (0.002 to 4 mM) for *K_m_* determination.

Methacrylyl-CoA hydratase activity was detected by the formation of 3-hydroxy-2-methylpropionyl-CoA from methacrylyl-CoA. The 20-μl reaction mixture contained 100 mM Tris-HCl (pH 7.8), 1 mM methacrylyl-CoA, and purified enzyme. The concentration of methacrylyl-CoA was varied (0.01 to 5 mM) for *K_m_* determination.

(*E*)-2-Octenoyl-CoA hydratase activity was detected by the formation of 3-hydroxyoctanoyl-CoA from (*E*)-2-octenoyl-CoA. The 20-μl reaction mixture contained 100 mM Tris-HCl (pH 7.8), 0.2 mM (*E*)-2-octenoyl-CoA, and purified enzyme. The concentration of (*E*)-2-octenoyl-CoA was varied (0.005 to 2 mM) for *K_m_* determination.

### Database search and phylogenetic analysis.

Query sequences for the database searches were obtained from NCBI database. The BLASTP searches were performed via NCBI BLAST server (http://www.ncbi.nlm.nih.gov/BLAST/) (41) and via the Integrated Microbial Genomes & Microbiomes system (https://img.jgi.doe.gov/m/) (42). The phylogenetic tree was constructed by using the maximum likelihood method and Jones-Taylor-Thornton (JTT) matrix-based model (43) in MEGA X (44). One hundred sixty-six amino acid sequences were involved in this analysis. All positions containing gaps were completely deleted. The GenBank accession numbers for the protein sequences are listed in Table S3.

### Mass spectrometric expression analysis.

Cell lysates were prepared for mass spectrometric (MS) analysis by filter-based reduction, alkylation, and tryptic digestion as described in reference 45 and measured at a concentration of 500 ng/μl. Samples were spiked with 47.6 fmol/μl yeast alcohol dehydrogenase 1 and 23.9 fmol/μl enolase 1. High-definition MS was performed using Synapt G2 Si ion mobility mass spectrometer coupled to M-Class UPLC (Waters Corp.) as described in reference 46 using a 90-min gradient (solvent system 100% water versus 100% acetonitrile, both containing 0.1% formic acid; trap column V/M Symmetry C18 100 Å 5 μm, 180 μm by 20 mm; reversed phase column HSS T3 1.8 μm, 75 μm by 200 mm; 3- to 4-μl injection volume, 3 technical replicates). Data were analyzed with ProteinLynx Global Server (Waters Corp.) using the *Metallosphaera* UniProt database containing the sequences for the spike proteins alcohol dehydrogenase and enolase 1 for protein identification.

### Other methods.

Protein was measured according to the Bradford method (47) using bovine serum albumin as a standard. *K*_m_ and *V*_max_ values were calculated by GraphPad Prism5 software. DNA sequence determination of purified plasmids was performed by Eurofins (Ebersberg, Germany). Sodium dodecyl sulfate-polyacrylamide gel electrophoresis (SDS-PAGE; 12.5%) was performed as described by Laemmli (48). Proteins were visualized using Coomassie blue staining (49). The purity of the recombinant protein was calculated by the determination of band intensities on SDS-gel using Image Lab software (Bio-Rad). Protein identification and quantification were performed at the IZKF Core Unit Proteomics Münster based on tryptic in-gel digestion and mass spectrometric analysis using Synapt G2 Si coupled to M-Class (Waters Corp.).

### Data availability.

All data are available in the main text or the supplemental material. The materials are available from the corresponding author upon reasonable request.
