# Supplementary material for: Convergent Evolution of a Promiscuous 3-Hydroxypropionyl-CoA Dehydratase/Crotonyl-CoA Hydratase in Crenarchaeota and Thaumarchaeota
Source: mSphere. 2021 Jan 20;6(1):e01079-20. doi: 10.1128/mSphere.01079-20 (PMC7845616; doi:10.1128/mSphere.01079-20)
Supplement: TABLE S2 [file mSphere.01079-20-st002.docx]

**Table S2.** Primers used in this study. The restriction enzymes used for the cloning are shown in parentheses and the corresponding restriction sites are underlined.

| **Primer** | **Sequence (5’-3’)** | **Used for** |
| --- | --- | --- |
| 2001_F | GCGGCCATATCGAAGGTCGTCATATGGAATTTGAAACAATAGAAAC (NdeI) | cloning of *msed_2001* |
| 2001_R | TCTCATGTTTGACAGCTTATCATCGATAAGCTTGTGGAGATCGTTTAAGTTCTCTTC (HindIII) |  |
| 0566_F | ACACATATGGATGTTATCCTTGAAAAGAG (NdeI) | cloning of *msed_0566* |
| 0566_R | TTAGGATCCGAGAAACCGACTGAGGACCATG (BamHI) |  |
| 0385_F | AATCATATGTCTCTAGTTCAGATTCGGG (NdeI) | cloning of *msed_0385* |
| 0385_R | AATAAGCTTTCATTCACCCCTGAAACTAGG (HindIII) |  |
| 1308_F | CTTGACGGATCCCATGTCACTAGTTAC (BamHI) | cloning of *nmar_1308* |
| 1308_R | GAAATAATCGATAGCTATTTCTTTGACTTGTTG (ClaI) |  |
| 2089_F | GAAGGTCGTCATATGCTCGAGATGGAGGACAAGC (XhoI) | cloning of *slip_2089* |
| 2089_R | GTTAGCAGCCGGATCCTTATTTGTCCTTGAATGCG (BamHI) |  |
